# Rhein methotrexate-decorated solid lipid nanoparticles altering adjuvant arthritis progression through endoplasmic reticulum stress-mediated apoptosis

**DOI:** 10.1007/s10787-023-01295-w

**Published:** 2023-08-01

**Authors:** Wessam M. El-Refaie, Mostafa S. Ghazy, Fady A. Ateyya, Eman Sheta, Mohanad Y. Shafek, Mahmoud S. Ibrahim, Mahmoud MA. Ismail, Mennatallah A. Gowayed

**Affiliations:** 1 Department of Pharmaceutics and Pharmaceutical Technology, Faculty of Pharmacy, Pharos University in Alexandria, Alexandria, Egypt; 2 Faculty of Pharmacy, Pharos University in Alexandria, Alexandria, Egypt; 3 Department of Pathology, Faculty of Medicine, Alexandria University, Alexandria, Egypt; 4 Department of Pharmacology and Therapeutics, Faculty of Pharmacy, Pharos University in Alexandria, Canal El- Mahmoudia Str., Smouha, Alexandria, Egypt

**Keywords:** Rhein, Methotrexate, Solid lipid nanoparticles, Adjuvant arthritis, Rheumatoid arthritis, Endoplasmic reticulum stress-induced apoptosis

## Abstract

Methotrexate (MTX) and diacerein (DIA) are two of the most potent disease-modifying anti-rheumatic drugs used for the treatment of rheumatoid arthritis (RA). DIA has reflected some GIT and hepatobiliary manifestations in numerous cases. It undergoes biotransformation in the liver into the active metabolite rhein (RH) which is characterized by its excellent anti-inflammatory activity and lower side effects. However, RH's hydrophobic nature and low bioavailability do not encourage its use in RA. The current study aims to use RH in combination with MTX in targeted solid lipid nanoparticles (RH-MTX-SLNs) for better effectiveness and shadowing light on its possible mechanistic pathways. RH-MTX-SLNs were prepared and assessed for their quality attributes. The effect of the formulation was assessed in-vivo in an adjuvant arthritis animal model investigating the role of the endoplasmic reticulum stress (ERS)-induced apoptosis. Results revealed that RH-MTX-SLNs were in the suitable nanosized range with high negative zeta potential indicating good stability. In-vivo, RH-MTX-SLNs significantly improved all measured inflammatory and arthritic markers, confirmed by electron microscopy and histology examination of the joints. Besides, the formulation was able to alter the ERS-mediated apoptosis. In conclusion, RH-MTX-SLNs can represent a promising therapeutic approach for RA showing significant anti-arthritic activity.

**Graphical abstract:**

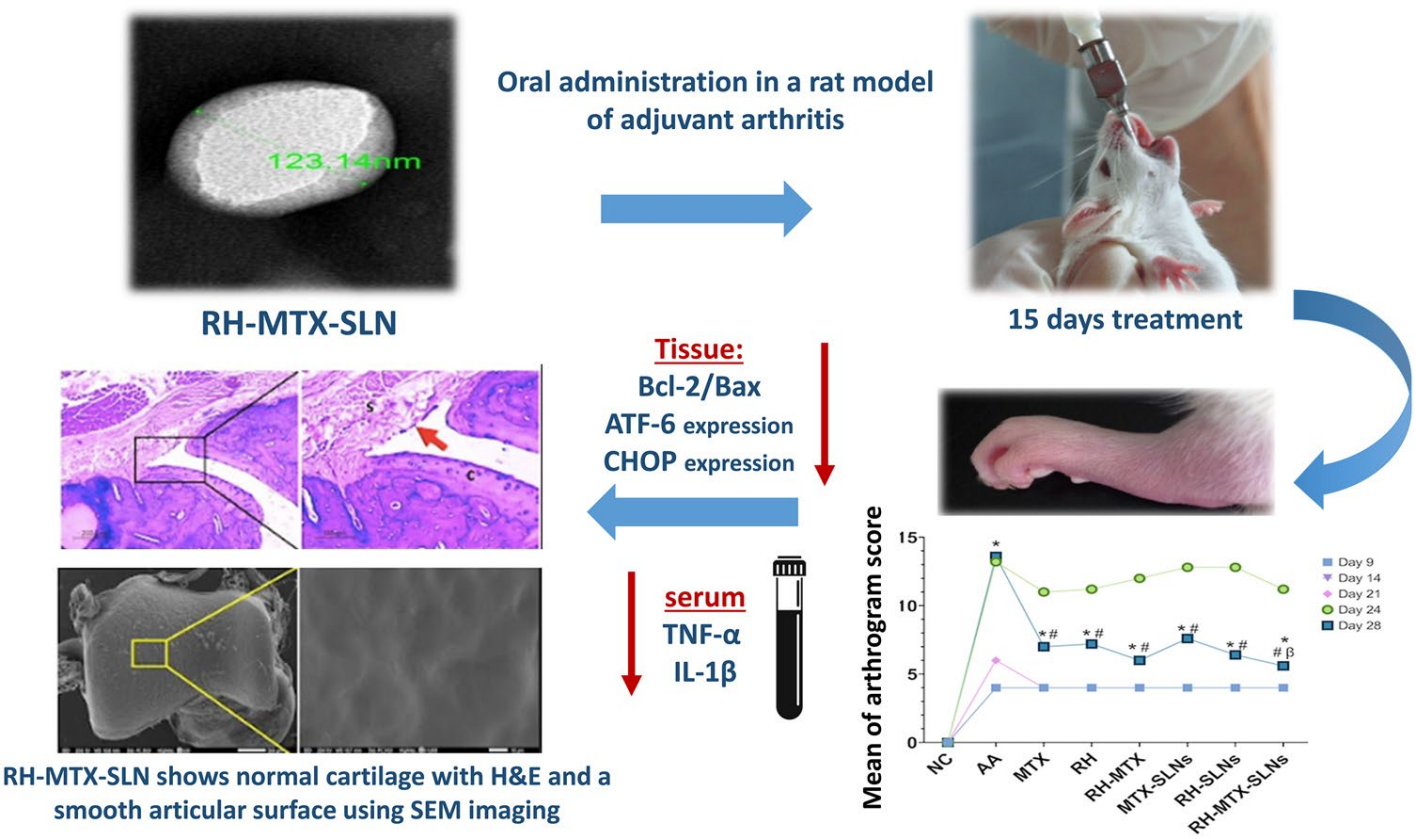

**Supplementary Information:**

The online version contains supplementary material available at 10.1007/s10787-023-01295-w.

## Introduction

Rheumatoid arthritis (RA) is a chronic autoimmune disease causing synovial inflammation and disability, especially in women, who show inferior disease activity scores than men (Sokka et al. 2009). RA cases are presenting with numerous similar symptoms like inflammation in the small joints of the lower and upper extremities as well as early morning pain and fatigue (Birch and Bhattacharya 2010). Conventional RA treatment is now limited to disease-modifying anti-rheumatic drugs (DMARDs) like leflunomide, sulfasalazine, and methotrexate (MTX), as well as biological DMARDs (bDMARDs) that inhibit cytokines, T- and B-cells. While they serve as antiarthritic and anti-inflammatory, numerous cases suffer from hepatotoxicity, hematological diseases, gastrointestinal and CNS manifestations (Buhroo and Baba 2006; Gaffo et al. 2006).

The trend toward herbal drugs has been expanding in the last few years (Li and Jiang 2018). The plant-derived diacerein (DIA) was used in research as an anti-inflammatory that reduces progressive joint destruction (Tamura et al. 2002; Louthrenoo et al. 2019). Besides its positive contribution to arthritis, DIA has reflected some GIT and hepatobiliary manifestations in numerous cases (Pavelka et al. 2016). Being a prodrug, it undergoes biotransformation in the liver into the active metabolite rhein (RH) which is characterized by its excellent anti-inflammatory as well as antitumor activity with lower adverse effects (Jain et al. 2014; Pavelka et al. 2016). In the last few years, a combination of a DMARD with DIA has shown promising effects in the treatment of early RA (Louthrenoo et al. 2016). Although RH has lower side effects compared to DIA, it shows poor solubility and oral bioavailability (Cong et al. 2012a). RH belongs to the biopharmaceutics classification system (BCS) class II of drugs, which have superior permeability but inadequate solubility. The unacceptable low water solubility of RH results in low bioavailability, which greatly limits its clinical application (Luo et al. 2019; Yao et al. 2021).

Nanocarriers have been of interest in the last few years because of their ability to deliver medicines to the target site inside the body, besides their surface can be modified by adding targeting ligands or other medicines for a precise delivery (Rawat et al. 2006). Lately, MTX over other DMARDs has been of interest to be incorporated in drug delivery nano vehicles for better bioavailability, enhanced solubility, controlled release, active targeting and hence, decreased toxicity (Choi et al. 2018). Thus, the current study aimed at combining RH with MTX in a nano vehicle aiming at enhancing their bioavailability and reducing MTX dose. Among colloidal nano-sized systems are the polymeric nanocarriers, liposomes, and solid lipid nanoparticles (SLN). SLN was selected for this study due to its merits as they contain lipids that are characterized by low toxicity and can incorporate lipophilic medicines such as RH. Also, the system is physically stable compared to other nanocarriers. Surveying the literature, no studies were conducted on utilizing nanotechnology for combining RH and MTX in the treatment of RA via the oral route.

Hence, the aim of this study is to formulate rhein-methotrexate SLN and assess its effect in the adjuvant arthritis (AA) animal model shadowing light on its possible influence on the anti-apoptotic signaling pathways. Knowing that impaired apoptosis of synovial fibroblasts is key in RA pathogenesis (Baier et al. 2003), and that activating transcription factor-6 (ATF-6) and C/EBP homologous protein (CHOP) are essential for the adaptive repair mechanism (Rahmati et al. 2018), make this mechanistic pathway attractive for the validation of the formulation success in AA.

## Methods

### Animals

A total of 48 male adult Sprague–Dawley rats (160–200 g, 7 weeks old) were used in this study. Rats were acquired from the animal house of Pharos University in Alexandria, Egypt, and kept under observation for 1 week preceding the experiment with free access to food and water. The animal procedures were permitted by the Research Ethical Committee of the Faculty of Pharmacy, Pharos University in Alexandria, Egypt (Approval No.: PU01202203273033) and comply with the ARRIVE guidelines, as well as the National Institute of Health for the care and use of laboratory animals.

### Chemicals and drugs

Rhein, methotrexate, Precirol Ato5 (glyceryl palmitostearate), Poloxamer 188, and lecinol (lecithin hydrogenated) were purchased from Baoji Guokang Biotechnology Co., LTD. Potassium chloride, sodium chloride; disodium hydrogen phosphate and potassium dihydrogen phosphate were acquired from Al-Nasr pharmaceutical Co., Cairo, Egypt. For induction of adjuvant arthritis, incomplete Freund’s adjuvant (Sigma Aldrich Co. MO, USA) and Mycobacterium butyricum (Difco Laboratories Co. NJ, USA) were purchased. All ELISA kits used were acquired from Elabscience (Texas, USA), rat Bcl-2 (B-cell Lymphoma) ELISA Kit Cat. No.: E-EL-R0096, rat Bax (Bcl-2 associated X protein) ELISA Kit Cat. No.: E-EL-R0098, rat TNF-α (Tumor Necrosis Alpha) ELISA Kit Cat. No.: E-EL-R2856, rat IL-1β (Interleukin 1 Beta) ELISA Kit Cat. No.: E-EL-R0012. The kit for RNA isolation was purchased from Thermo Fisher Scientific (Massachusetts, USA, Cat. No. 12183555) and the kit for reverse transcription was bought from Qiagen (USA, Cat. No. 204772). All other chemicals were of analytical grades.

### Preparation of RH-MTX solid lipid nanoparticles

RH-MTX SLNs were prepared by the modified high shear homogenization method (El-Salamouni et al. 2015) with little modifications. The lipid phase, Precirol ATO5 (2%*w*/*w*) and Lecinol (2%*w*/*w*) was melted at 80 ± 0.5 °C. RH (1 mg/ml) and MTX (15 µg/ml) were dissolved in a minimum amount of ethanol: acetone (1:1) and added to the lipid phase. The aqueous phase containing Poloxamer 188 (2.5% *w*/*v*) was heated to the same temperature. Then, it was poured to the lipid phase and subjected to 5 min hot homogenization at 20,000 rpm (Ultra Turrax T25, IKA, Germany) followed by 10 min sonication. The obtained SLNs were cooled at room temperature 25 °C then refrigerated at 4 °C. Using this method placebo SLNs, RH-SLNs, and MTX-SLNs were prepared.

### In-vitro characterization of the prepared SLNs

#### Determination of particle size, polydispersity index, and zeta potential

Dynamic light scattering (DLS) technique was used for the determination of particle size (PS), zeta potential (ZP) and polydispersity index (PDI) using Zetasizer (Nano ZS, Malvern, UK). Samples were appropriately diluted with deionized distilled water before measurement.

#### Determination of % entrapment efficiency

The prepared SLNs were centrifuged at 15,000 rpm for 30 min at 4 °C. Supernatants containing the unentrapped drug were withdrawn and dissolved in acetone: ethanol (1:1) (Feng et al. 2017). Drug concentration was measured spectrophotometrically at the predetermined λ_max_ for each drug (259 nm for RH and 301 nm for MTX). Samples were analyzed in triplicates and the data are presented as mean ± SD.

% Entrapment efficiency was determined according to the following equation (Khalil et al. 2022):

1 $${\text{\% }}\,{\text{Entrapment efficiency }} = \frac{{{\text{total drug}} - {\text{unentrapped drug}}}}{{\text{Total drug}}}\,*100$$

#### Transmission electron microscopy (TEM)

The morphology of the developed SLNs was investigated using TEM (190 JOEL, CX, Japan). Samples drop of the nanoparticle dispersion were diluted with deionized water and a drop was spread on a carbon-coated grid. Then they were stained with uranyl acetate and air-dried under ambient conditions prior to the microscopic examination (Komeil et al. 2022).

#### Fourier transform infrared spectroscopy (FT-IR)

FTIR spectra of RH, MTX, Precirol ATO5, Lecinol, Poloxamer 188, physical mixture, and SLNs were investigated. Each sample spectrum was determined over the spectral region of 4000–700 cm^−1^ through an Affinity-1 spectrophotometer (Cary 630 FTIR Agilent Technologies, Malaysia).

#### In-vitro drug release study

The *in-vitro* release profiles of RH and MTX from the prepared SLNs were determined, as compared to RH and MTX suspension, using the dialysis bag method VISKING dialysis tubing molecular weight cut off 12–14 kDa (SERVA, electrophoresis, Germany). 2 ml of each formulation were filled into dialysis bags that were immersed in 50 ml PBS at pH 6.8 with 20% ethanol (Feng et al. 2017). They were placed in a horizontal shaking water bath at 100 rpm and 37 ± 0.5 °C. At predetermined time intervals (1, 2, 3, 4, 5, 6, 7, 8 and 24 h), samples were withdrawn and immediately replaced with the same volume of fresh warmed release medium. The amount of drug was measured spectrophotometrically at λ_max_ 301 nm for MTX and 259 nm for RH. Samples were analyzed in triplicates and the results were presented as mean ± SD.

#### Stability study

The prepared SLNs were stored in glass containers at 4 °C for 3 months. Formulations were visually inspected for any physical change in addition to monitoring changes in particle size, ZP, PDI and % EE upon storage.

### Animal experiment

#### Induction of adjuvant arthritis

To acquire an AA model, rats were injected with 0.1 ml suspension of heat-killed Mycobacterium butyricum in an incomplete Freund’s adjuvant (12 mg/ml) intradermal at the base of the tail (Gowayed et al. 2020). Rats were observed for 13 days after induction for the development of AA.

#### Experimental design

On day 13 of AA development, rats were assigned into eight groups of 6 rats each. Group 1: (NC) non-arthritic healthy control rats obtaining oral saline. Group 2: AA rats obtaining oral saline. Group 3: AA rats treated with MTX (1 mg/kg/week; i.p.) as a reference dose (Gowayed et al. 2020). Group 4: AA rats treated with an oral dose of RH (10 mg/kg). Group 5: AA rats treated with an oral dose of MTX and RH solution (RH-MTX; 150 μg/kg and 10 mg/kg, respectively). Group 6: AA rats treated with an oral dose of MTX-SLNs (150 μg/kg). Group 7: AA rats treated with an oral dose of RH-SLNs (10 mg/kg). Group 8: AA rats treated with an oral dose of RH-MTX-SLNs 10 mg/kg and 150 μg/kg, respectively.

Tested drugs were administered 3 times per week (except MTX) for 15 days (day 14–day 28 from adjuvant injection).

#### Assessment of arthritis progression

The hind paw swelling of the ankle tibiotarsal joint width was measured by caliper in all groups on day 0 before induction followed every other day until day 28 of the experiment (Gowayed et al. 2020). The acuteness of arthritis was also scored on a 4-point scale **(**Table 1**)** using the Arthrogram scoring system (Refaat et al. 2013). Arthrogram scores were assessed on days 9, 14, 21, 24, and 28. The sum scores for all four limbs were calculated with a maximum possible score of 16 per rat and the mean score was presented.

**Table 1:** Arthrogram scoring system

Score	Interpretation
0	Normal
1	Slight oedema of the small digital joints
2	Oedema of the digital joints and footpad
3	Gross oedema of the entire footpad below the ankle or wrist
4	Gross oedema of the entire footpad including the ankle joint or wrist joint

#### Serum parameters

On day 29 of the study, an overdose of phenobarbital (200 mg/kg) (Gowayed et al. 2020) was used to euthanize the rats. A laparotomy incision was made for blood collection from the posterior vena cava. Serum levels of TNF-α and IL-1β were determined using ELISA kits according to manufacturer instructions.

#### Tissue parameters

At the end of the experiment, on day 29, the tibiotarsal joint of the right hind paw was and kept at − 80 °C till analysis. The tibiotarsal joint of the left hind paw was detached and washed with ice-cold saline. Four of the left hind paws (per group) were kept in formalin (10%) for further histopathological examination, while the two other left hind paws were kept in glutaraldehyde 4% for examination under the scanning electron microscope (SEM).

Synovial tissue was removed from the joint of the right hind paw and divided into two aliquots. One aliquot was used to determine the Bcl-2 and Bax amount using the ELISA kit according to the manufacturer’s instructions.

Another aliquot was used to extract the total RNA using TRIzol Plus RNA Purification kit for the assessment of gene expression by means of qRT-PCR techniques (according to the manufacturer’s instructions). Reverse transcription of total RNA into cDNA was done using Rotor-Gene RT Mix Kit (according to the manufacturer’s instructions).

#### Determination of gene expression in synovial tissues

The produced cDNA was used to measure the joint expression ATF-6 and CHOP genes by Rotor-Gene Q qPCR using QuantiTect SYBR Green PCR Master Mix. The PCR amplification was initiated with a denaturation step (5 min at 95 °C) and then amplified by 40 PCR cycles: Denaturation (95 °C for 5 s), annealing (60 °C for 10 s) and extension (60 °C for 10 s). Values of the threshold cycle (Ct) were defined by Rotor-Gene Q-Pure Detection (version 2.1.0). The change in mRNA level for each gene in the sample was concluded using the 2^−ΔΔCt^ method and normalized using the reference gene β-actin. Primer sequences used for gene expression are shown in Supplementary file 1. Online Resource 1.

#### Scanning electron microscopy

The tibiotarsal joints fixed in glutaraldehyde were washed in phosphate buffer and dehydrated in grades of alcohol. They were then dried and mounted on stages to be assessed using a JSM-IT200 scanning electron microscope (JEOL, Massachusetts, USA) at 80 kV. The surface ultrastructural characteristics of the cartilage covering the lower end of the tibia were then evaluated and photographed.

#### Histopathological study

The stored joint samples in formalin were cut and embedded in paraffin blocks; sliced sections (5 μm) were obtained and stained with hematoxylin–eosin (H&E) for the evaluation of synovial proliferation, inflammatory infiltration, and degenerative changes in the joints. Changes were graded according to Koizumi et al. as mentioned in Table 2. At least 3 fields per joint were assessed. The grades were then summed up into a score out of 9 (Koizumi et al. 1999).

**Table 2:** The scoring system of the joints according to Koizumi et al. (Koizumi et al. 1999)

	Mild (grade 1)	Moderate (grade 2)	Sever (grade 3)
Synovial proliferation	3–4 layers of synoviocytes	5–7 layers	> 7 layers or papillae
Inflammation	occupying < one-third of × 200 field	Occupying one to two theirs of × 200 field	occupying > two-thirds of × 200 field
Degenerative changes of joint	Minor destruction of cartilage and subchondral bone	Clear loss of cartilage and involvement of 30% of underlying bone	Total loss of articular cartilage and involvement of > 30% of underlying bone

#### Statistical analysis

For parametric data, values are described as means ± S.D (*n* = 6). Data were analyzed using one-way analysis of variance (ANOVA), followed by the Tukey multiple comparison post hoc test. For nonparametric data results were analyzed by Kruskal–Wallis Test, followed by Dunn's post hoc test. The differences were significant at *p* < 0.05. All statistical analysis and graphs were presented using the Prism computer program (GraphPad Software Inc. V9, San Diego, CA, USA).

## Results

### Preparation and characterization of RH-MTX SLNs

Four SLNs formulations namely, placebo SLNs, RH-SLNs, MTX-SLNs, and RH-MTX-SLNs were prepared using a modified high-shear homogenization method followed by sonication. They were investigated for the different in-vitro quality attributes and pharmacological activity. In-vitro characterization of SLNs was based on particle size, poly-dispersity index analysis (PDI), zeta potential, entrapment efficiency (% EE), morphological examination with TEM, FTIR, and in-vitro drug release.

#### Particle size, zeta potential, PDI, and entrapment efficiency

As shown in Table 3, all the prepared SLNs were in the nanosize range with good PDI < 0.4. A significant (*P* < 0.05) size increase was observed upon drug loading. High negative zeta potential was obtained for all formulations with insignificant (*P* > 0.05) differences between them. MTX-loaded SLNs showed slightly higher zeta potential. Generally, a high % EE was observed for both drugs in the prepared RH-SLNs and MTX-SLNs. An insignificant (*P* > 0.05) decrease in the % EE was noticed upon combining both drugs in RH-MTX-SLNs.

**Table 3:** Physicochemical characteristics of the prepared SLNs at zero time and after storage for 3 months at 4 °C

Code	Particle size (nm)		PDI		zeta potential (mV)		%Entrapment efficiency			
	Zero time	3 months	Zero time	3 months	Zero time	3 months	Zero time		3 months	
							MTX	RH	MTX	RH
Placebo-SLNs	136.53 ± 7.68	141.24 ± 5.31	0.36 ± 0.05	0.29 ± 0.08	− 44.60 ± 1.04	− 43.24 ± 2.10	–	–	–	–
MTX-SLNs	182.33 ± 2.83	186.52 ± 3.14	0.30 ± 0.01	0.34 ± 0.04	− 46.66 ± 0.75	− 44.97 ± 1.56	90.45 ± 2.14	–	88.78 ± 4.26	–
RH-SLNs	150.67 ± 6.96	153.14 ± 7.21	0.32 ± 0.08	0.36 ± 0.02	− 43.50 ± 2.48	− 45.23 ± 3.25	–	95.62 ± 1.07	–	94.87 ± 0.74
RH-MTX SLNs	192.37 ± 3.07	196.94 ± 4.56	0.37 ± 0.02	0.39 ± 0.01	− 47.43 ± 1.90	− 45.14 ± 2.64	88.21 ± 3.10	93.02 ± 0.95	87.85 ± 2.49	92.35 ± 1.25

Results are represented as mean ± SD (*n* = 3)

#### Fourier transform infrared spectroscopy

**Fig. 1:**
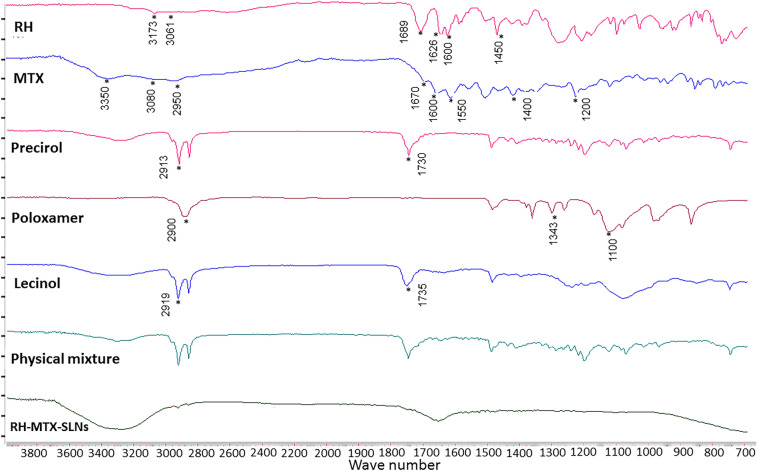
FTIR spectra of RH, MTX, Precirol, Lecinol, Poloxamer, Physical mixture and RH-MTX SLNs

Figure 1 shows the FTIR spectra of each of the formulation ingredients, RH, MTX, physical mixture, and RH-MTX-SLNs. The obtained IR spectrum of RH showed characteristic peaks at 1450 cm^−1^ and 1626 cm^−1^ that are assigned to the stretching vibrations of aromatic C = C and C = O, respectively. In addition to the characteristic peaks observed at 1689 cm^−1^ (C = O stretch, COOH), 3061 cm^−1^ (O–H stretch, COOH) and 3173 cm^−1^ for aromatic O–H vibrations (Kaur et al. 2014). MTX IR spectrum shows a broad characteristic absorption band at 3350 cm^−1^ corresponding to O–H stretches from the carboxyl group. At 3080 cm^−1^, the primary amine group, N–H, stretching vibrations were revealed. In addition, C–H vibration from CH_3_ group appeared at 2950. The splinted band at 1670–1600 cm^−1^ corresponds to C = O stretching from the carboxylic group and the amidic group. amidic N–H band appears at 1550–1500 cm^−1^ overlapping with the stretching vibrations of aromatic C = C. The carboxylic group band appears in the range of 1400–1200 cm^−1^, corresponding to the –C–O stretching (Bajas et al. 2021).

Precirol^®^ IR spectrum showed a characteristic peak at 1730 cm^−1^ corresponding to C = O stretching and 2913 cm^−1^ for C-H stretching which are characteristic of glyceryl palmitostearate. Lecinol characteristic peaks appeared at 2919 cm^−1^ and 2921 cm^−1^ for methylenic C–H group stretching, and at 1739 cm^−1^ and 1735 cm^−1^ for C = O group stretching (Youssef et al. 2020). The IR spectrum of poloxamer 188 revealed absorption peaks at 3485 cm^−1^ for O–H stretching, 2900 cm^−1^ for aliphatic C-H stretch, 1343 cm^−1^ (in-plane O–H bend) and 1100 cm^−1^ for C–O stretch (Manikandan et al. 2013).

The physical mixture spectrum showed no peaks for the drugs. The IR spectrum of the formulation showed a stretching broad peak at 3200–3600 cm ^−1^ observed corresponding to O–H for water which makes the external phase (Youssef et al. 2020). In addition, it did not show the characteristic drug peaks. However, some functional groups of the lipid excipients were observed.

#### Transmission electron microscope

**Fig. 2:**
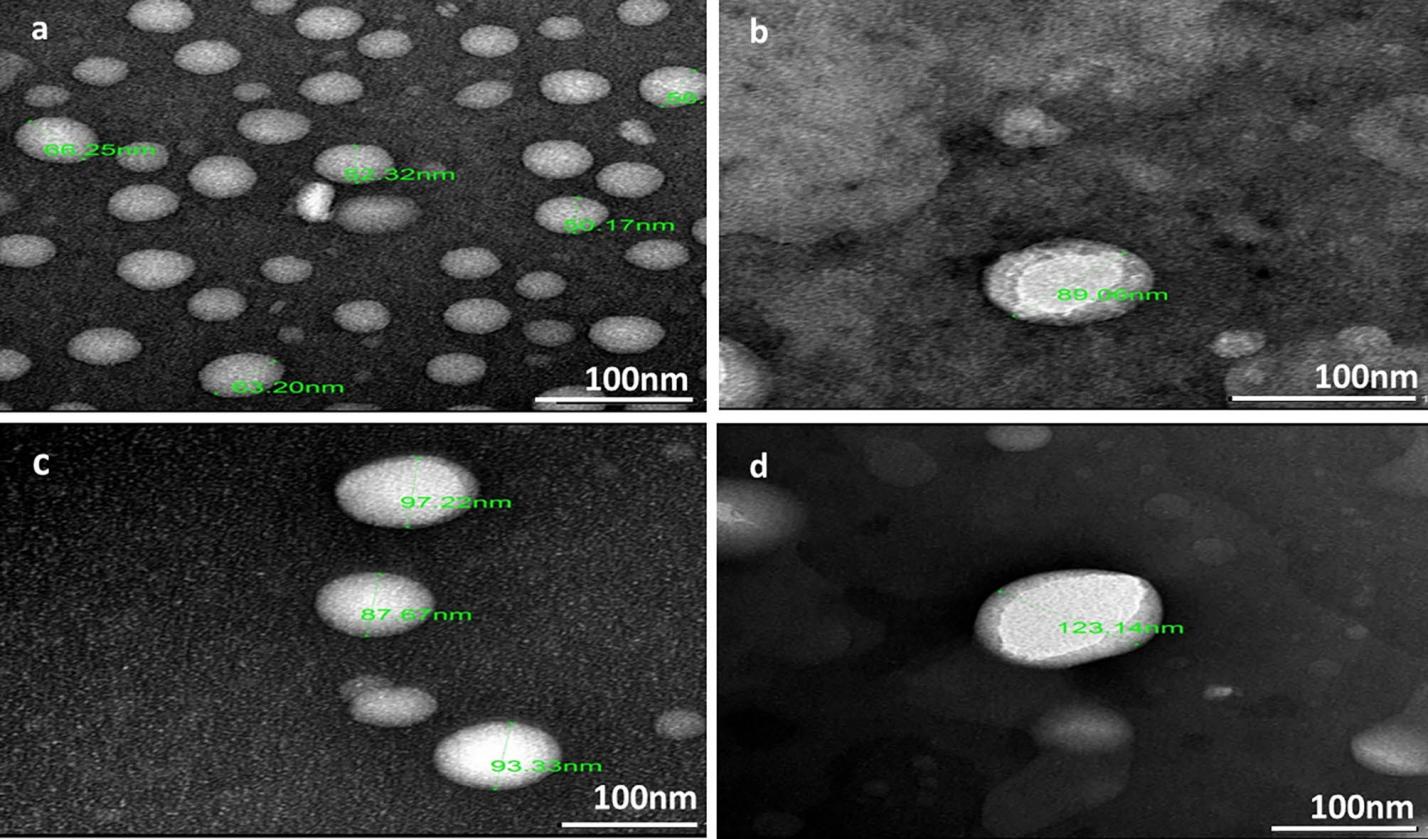
Transmission electron micrographs of **a** Placebo SLNs, **b** MTX-SLNs, **c** RH-SLNs, and **d** RH-MTX-SLNs

TEM micrographs (Fig. 2) indicated the smooth surface of all the prepared SLNs with no aggregation. MTX-SLN (Fig. 2b) showed a dense layer surrounding the particles that are revealed also in RH-MTX-SLN (Fig. 2d).

#### In-vitro drug release

**Fig. 3:**
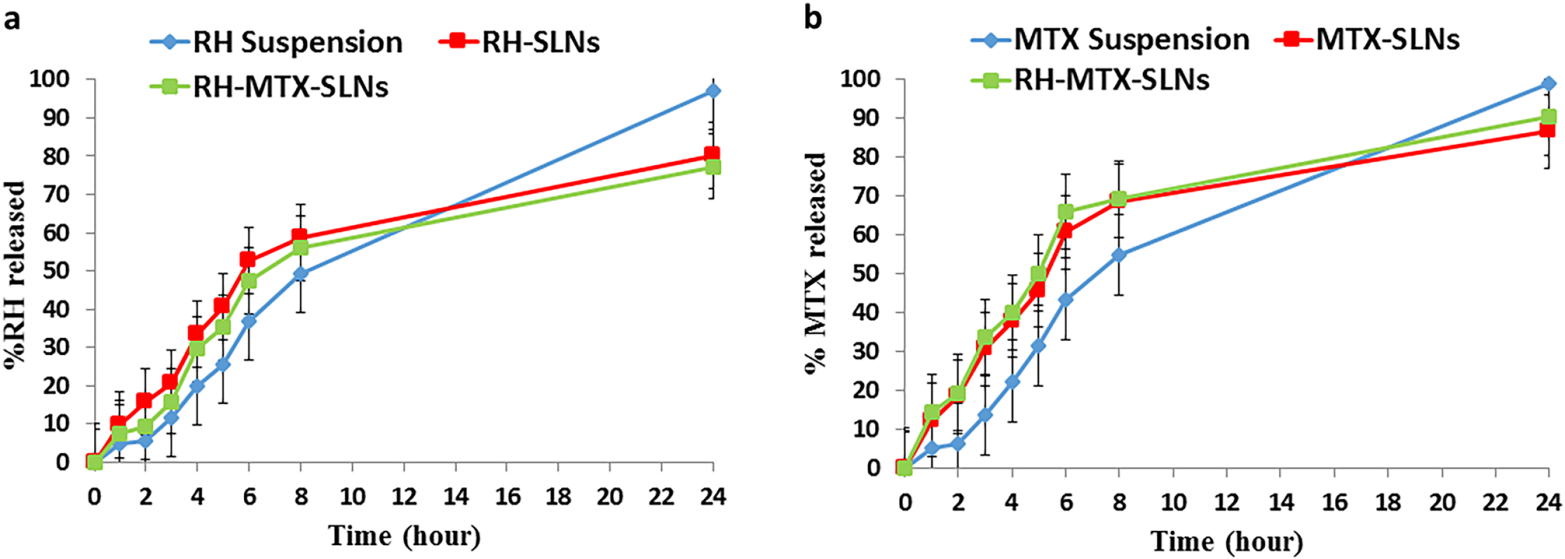
In-vitro release behavior of **a** RH from RH-suspension, RH-SLNs and RH-MTX-SLNs, **b** MTX from MTX-suspension, MTX-SLNs, and RH-MTX-SLNs, using dialysis bag technique in PBS PH (6.8) with 20% ethanol at 37 ± 1 °C for 24 h. The error bars indicate the corresponding standard deviations

As shown in Fig. 3, higher RH and MTX release was observed from the prepared SLNs compared to the corresponding drug suspension. However, the release obtained from the prepared SLNs was sustained. At 2 h, 18.42 ± 2.56% and 19.52 ± 5.01% MTX were released from MTX-SLN and RH-MTX-SLNs; respectively. For RH, 15.72 ± 3.14% and 9.41 ± 1.06% were released at 2 h from RH-SLNs and RH-MTX-SLNs, respectively. A higher release rate was observed from MTX compared to RH. After 3 h RH release was 20.72 ± 4.28% from RH-SLNs and 15.94 ± 2.34% from RH-MTX-SLNs. On the other hand, MTX release after 3 h was 30.67 ± 3.54% from MTX-SLNs and 33.61 ± 1.68% from RH-MTX-SLNs.

### Stability study

The prepared SLNs showed good stability for 3 months at 4 °C as shown in Table 3. Particle size, PDI, ZP and % EE didn't demonstrate any significant (*P* > 0.05) change upon storage. In addition, no aggregation, or changes in the translucence of the formulation was observed.

### Effect on arthritis progression

**Fig. 4:**
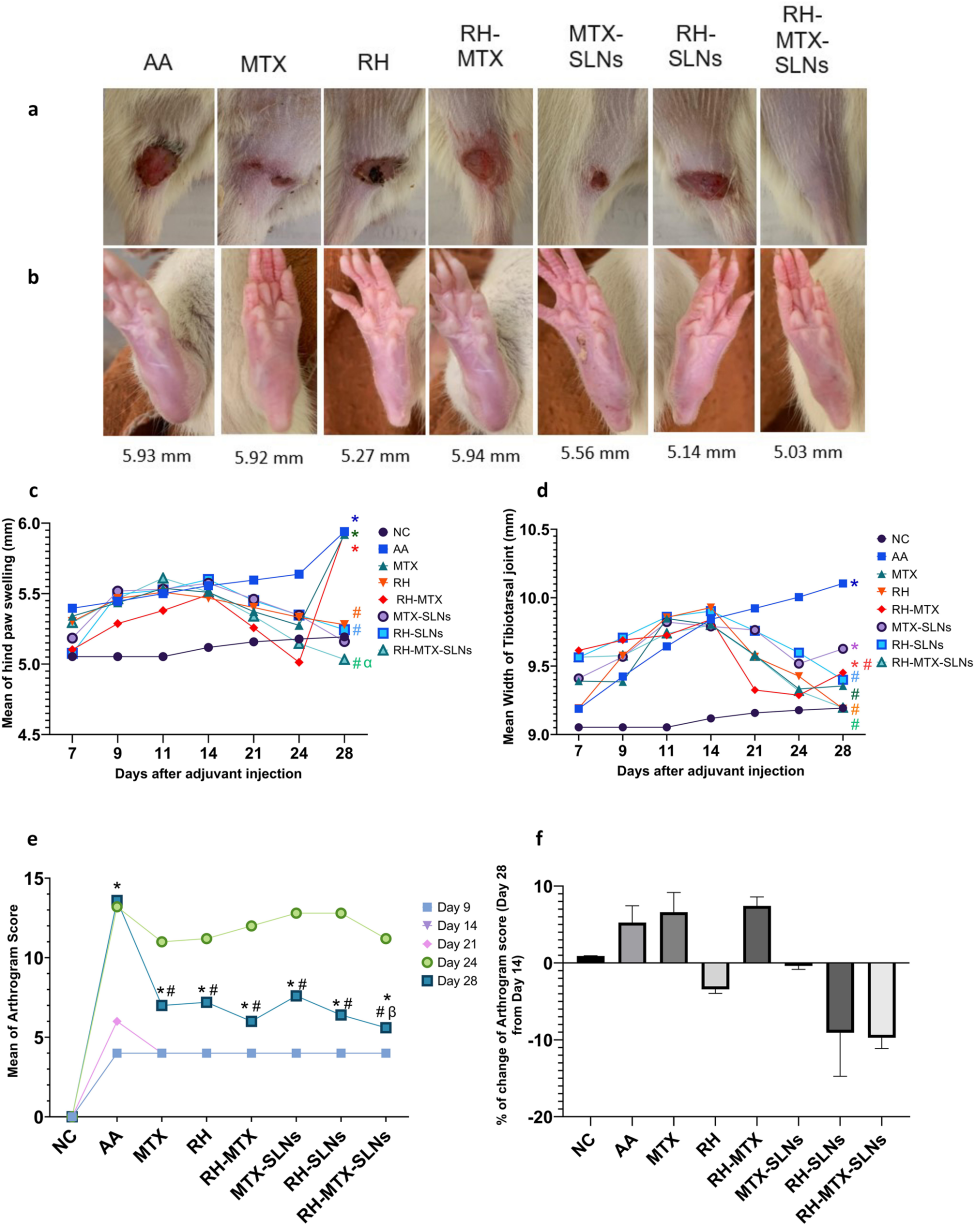
Effect on Arthritis progression. **a** Representative photographs showing ulceration at the base of the tail, **b** representative photographs showing hind paw swelling, **c** mean of hind paw swelling (mm), **d** mean width of the tibiotarsal joint (mm), **e** mean of arthrogram score and **f** % change of arthrogram score of day 28 from day 14 after induction. Data are presented as mean ± SD, *n* = 6. Symbols show a comparison to * negative control (NC), # positive control (AA), α combination of Methotrexate and rhein in solution form (RH-MTX), β Methotrexate loaded on Solid Lipid Nanoparticles (MTX-SLNs), using ANOVA (*p* < 0.05). MTX: methotrexate solution, RH: rhein solution, RH-SLNs: rhein loaded on solid lipid nanoparticles, RH-MTX-SLNs: combination of methotrexate and rhein loaded on solid lipid nanoparticles

The arthritis progression rate of RH-MTX-SLNs was compared with all treated groups and the untreated ones after 28 days from the start of the treatment. On day 28, there was almost no ulceration at the base of the tail in the RH-MTX-SLNs group compared to other groups (Fig. 4a). A great reduction in the hind paw swelling of RH-MTX-SLNs treated rats was observed (5.34 mm), showing a significant difference from untreated AA rats (5.03 mm, *p* = *0.0003*), MTX treated rats (5.92 mm, *p* < *0.0001*) and RH-MTX treated ones (5.94 mm, *p* < *0.0001*), Fig. 4b, c. The mean width of the tibiotarsal joint was measured as well on day 28 for RH-MTX-SLNs treated rats (9.20 mm) showing a significant difference compared with the untreated group (10.13 mm*, p* = *0.005*), while no difference was seen compared to negative control rats Fig. 4d. The mean of the Arthrogram score (Fig. 4E) also reflects the progression of arthritis from day 9 to day 28, where on day 28 RH-MTX-SLNs rats showed significant difference compared to MTX-SLNs rats (*p* = *0.0017*), and the untreated AA group (*p* < *0.0001*). The % of change of Arthrogram score (day 28 from day 14) has confirmed that the effect of different treatments showed the highest change in RH-MTX-SLNs by -9.733%, while RH-SLNs was second showing − 9.083% (Fig. 4f).

### Effect on inflammation

**Fig. 5:**
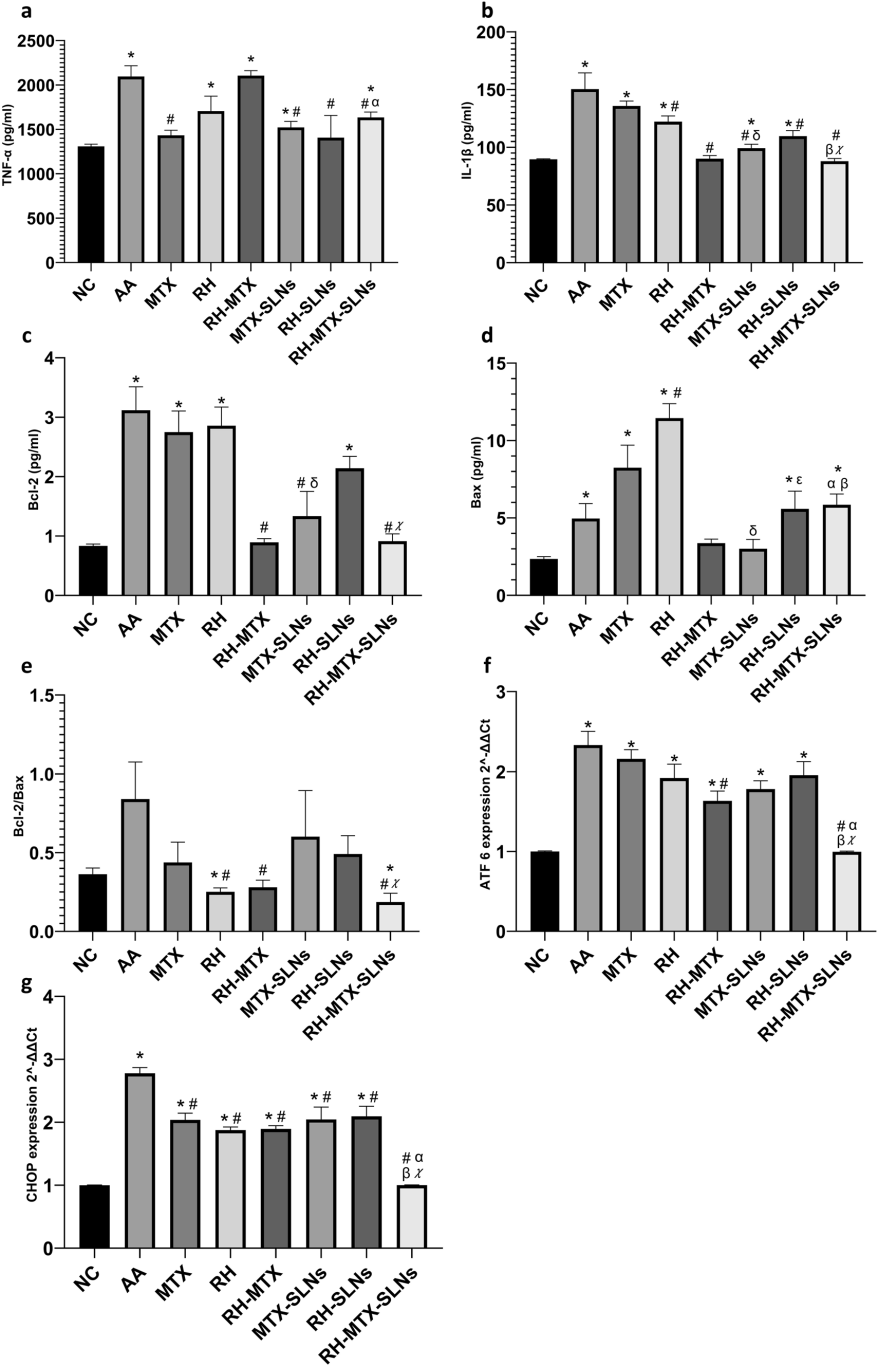
Effect on inflammation and endoplasmic reticulum stress-induced apoptosis. **a** Tumor necrosis factor-α (TNF-α, pg/ml), **b** interleukin-1 (IL-1β, pg/ml), **c** B-cell Lymphoma (Bcl-2, pg/ml), **d** Bcl-2 associated X protein (Bax, pg/ml), **e** Bcl-2/Bax ratio **f** ATF6 expression and **g** CHOP expression. Data are presented as mean ± SD, *n* = 6. Symbols show a comparison to * negative control (NC), # positive control (AA), δ methotrexate solution (MTX), α combination of methotrexate and rhein in solution form (RH-MTX), β methotrexate loaded on solid lipid nanoparticles (MTX-SLNs), χ rhein-loaded on solid lipid nanoparticles (RH-SLNs) using ANOVA (*p* < 0.05). RH: rhein solution, RH-MTX-SLNs: combination of methotrexate and rhein loaded on solid lipid nanoparticles

Induction of adjuvant arthritis triggered the inflammatory cascade (Fig. 5), presented as increased levels of TNF-α and IL-1β. All treated groups have shown significant reduction in TNF-α and IL-1β levels compared to AA rats, except the RH-MTX TNF-α level (Fig. 5a). While RH-MTX-SLNs showed a significant decrease in TNF-α levels from AA rats and RH-MTX treated rats (22% decrease), it showed no difference to, MTX-SLNs (27% decrease, p = 0.245) and RH-SLNs (32% decrease, *p* = 0.396) treated rats.

However, treatment with RH-MTX-SLNs showed the most significant decrease in IL-1 β levels (Fig. 5b) compared to AA rats (*p* < 0.0001), MTX (*p* < 0.0001), RH (*p* < 0.001), MTX-SLNs (*p* = 0.019) and RH-SLNs (*p* = 0.0024) treated rats, showing no difference to negative control rats and RH-MTX treated rats.

### Effect on endoplasmic reticulum stress-induced apoptosis

Figures 5c–e come to show an AA-related inhibition of apoptosis, where it showed significant elevation of both, the anti-apoptotic parameter (c) Bcl-2 and the apoptotic marker (d) Bax, compared to negative control rats. Calculating the Bcl-2/Bax ratio in AA rats (e) comes in favor of the Bcl-2 showing elevation. All treated groups showed a decreased Bcl-2/Bax ratio to a different extent, inducing apoptosis. Post-treatment with RH whether alone or combined with MTX in solution RH-MTX or RH-MTX-SLNs showed the most significant decrease in Bcl-2/Bax ratio compared to AA rats (*p* = 0.0326, *p* = 0.0421 and *p* = 0.0225, respectively). Examining markers of ERS, all treated groups were able to decrease the expression of ATF6, the sensor of ERS response, and the apoptogenic CHOP (Fig. 5f, g). Interestingly, the RH-MTX-SLNs were able to do a sharp decrease in the expression of ATF6 (*p* < 0.000) and CHOP (*p* < 0.0001) compared to AA rats, showing no difference from negative control, showing a relation to the decreased Bcl-2/Bax ratio in the apoptosis signaling pathway.

### Electron microscopy evaluation

**Fig. 6:**
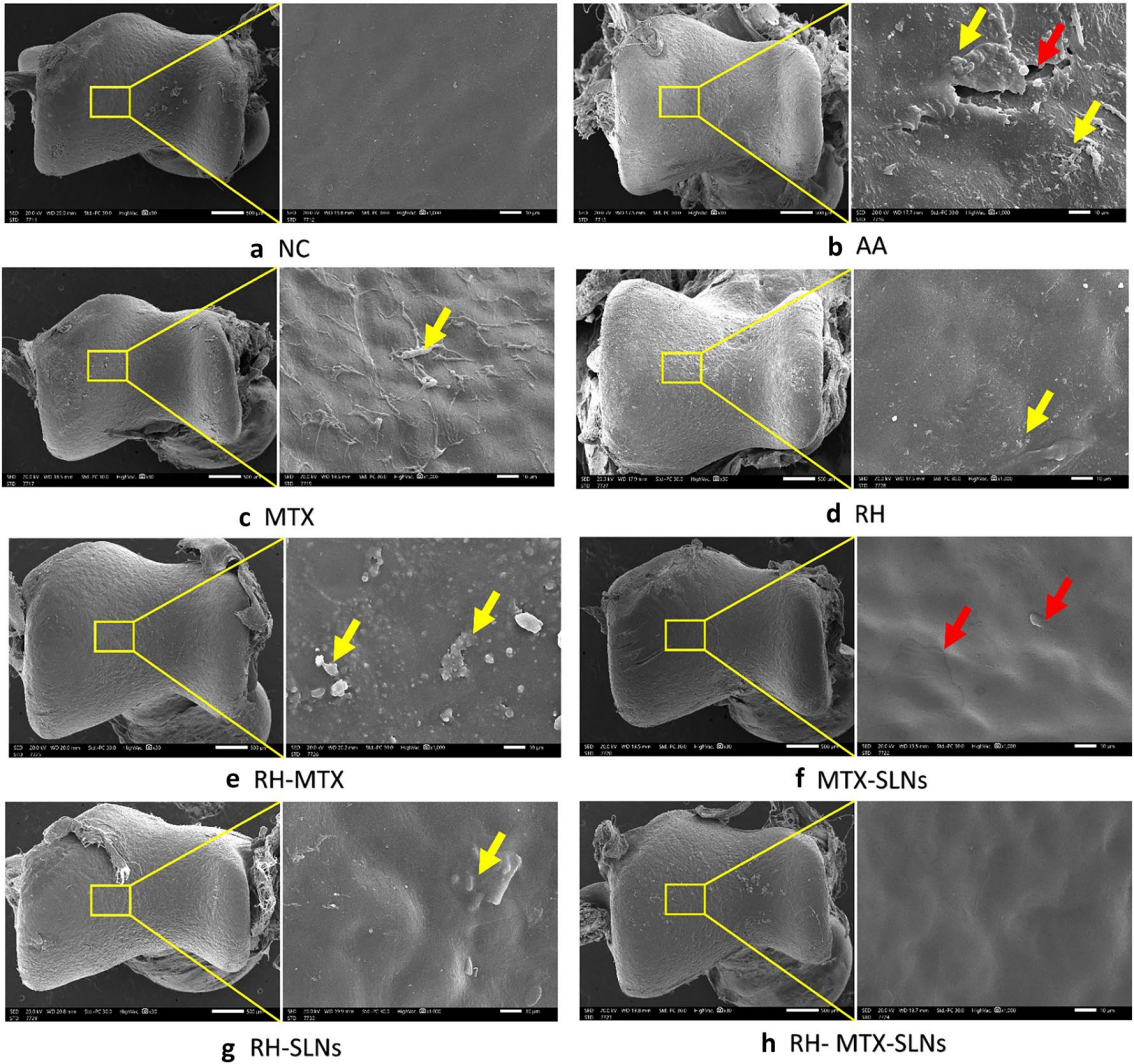
Scanning electron microscopy of the articular surface of the lower tibia of the hind paw in different studied groups (low power, × 30, scale bar 500 µm, high power × 1000, scale bar 10 µm): Normal control **a** showed smooth articular surface, whereas the adjuvant arthritic group **b** showed evident surface irregularities with fissuring (red arrow) and multiple deposits (yellow arrow). Those changes were reversed in different treated groups (C-H) with the best effect in the combined MTX RH SLN group. Red arrow: break or loss, yellow arrow: deposits

Scanning microscopy of the surface articular cartilage of normal control showed a smooth articular surface. In contrast to the AA model which was irregular and rough with multiple cracks and fissures. Also, fibrillations and deposits of different sizes were noted. Improvement of the articular surface was seen with different treated groups. Some fibrillations and partial roughness were still noted in MTX, and RH groups. Improvement was minimal in the combined group. Whereas the addition of SLN particles augmented the effect of MTX and RH in the restoration of the joint cartilage. However, the cartilage surface was smooth back again and close to normal when the combined therapy RH-MTX was loaded on SLNs (Fig. 6).

### Histopathology

**Fig. 7:**
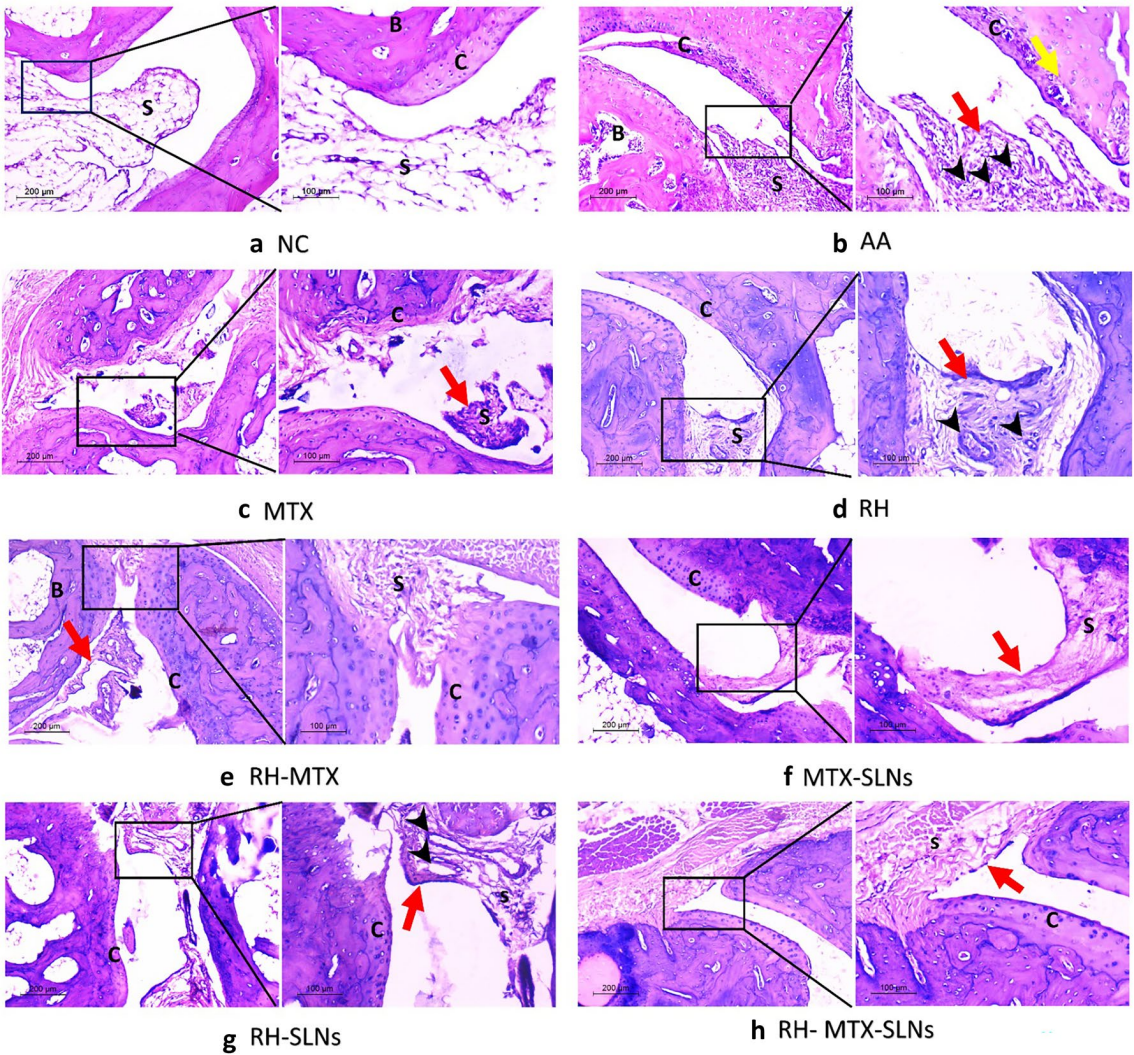
H&E stained sections of the hind paw of different studied groups (low power × 100, higher power × 200): **a** Normal control shows a thin synovial surface without inflammation or hyperplasia (S). The articular cartilage **C** is thick and smooth, and the underlying bone **B** is histologically free. **b** Adjuvant arthritis model showing hyperplastic papillary synovium (red arrow) with severe inflammation (S) and congested vessels (black arrow). The articular surface **C** is thinned out and irregular showing degenerated chondrocytes. Underlying bone shows hypercellular bone marrow **B** and bone cysts (yellow arrow). **c** MTX-treated group shows residual severe papillary hyperplasia (red arrow) of the synovium with moderate inflammation (S). The cartilage surface is still irregular **C**. **d** RH treated group showing residual moderate hyperplasia of the synovium (red arrow) with moderate inflammation (S). Articular cartilage (C) is thinned out with minor irregularities. **e** Combined RH-MTX treatment shows moderate hyperplasia of the synovium (red arrow) with minimal inflammation (S). Irregularities and erosions are still seen on the cartilaginous surface (C). **f** MTX-SLN treatment shows minimal hyperplasia (red arrow) and inflammation (S) of the synovium. Minor irregularities of the cartilage surface are also seen (C). **g** RH-SLNs group shows mild hyperplasia (red arrow) and inflammation (S). Cartilage is restored with minor irregularities (C). **h** The best effect was seen with the RH-MTX-SLNs group, where the synovium was not hyperplastic (red arrow) or inflamed (S) and the cartilage was restored (C). Red arrow: synovial lining, S = synovium, C = cartilage, B = bone, black arrow = blood vessels, yellow arrow = bone cysts

The normal control group showed thin synovium with no inflammation or proliferation. The articular cartilage was thick with a smooth surface. The AA model showed notable pathologic changes in rheumatoid arthritis in contrast to the normal joint. The synovium was severely hyperplastic, hypertrophied, and papillary-like. It was heavily infiltrated with mononuclear inflammatory cells. Congested vessels were commonly seen. The articular surface was thinned out and irregular with areas of erosion and total loss. The underlying bones showed severe involvement with hypercellular bone marrow and multiple bone cysts. Treated groups with different drugs showed different degrees of improvement. MTX showed minimal hyperplasia of synovium. The inflammation was moderate with less congestion. However, the articular cartilage was still thinned out with residual erosions. The improvement was slightly better in the group treated with RH. Meanwhile, the combined RH-MTX did not show a good effect revealing moderate synovial hyperplasia and residual inflammation. Adding drugs to the SLNs formula increased their effect, especially on synovial hyperplasia. However, the best effect was seen when both drugs were loaded on SLNs. In this RH-MTX-SLNs group, the inflammation was absent to minimal in synovium which was lined by a thin layer of synoviocytes with no hyperplasia. The articular cartilage was restored with the regeneration of chondrocytes and no erosions were seen (Fig. 7 and Table 4).

**Table 4:** Effect of the different treatments on the joint score

	Synovial proliferation	Inflammation	Degenerative changes	Total (out of 9)
NC	0	0	0	0
AA	3	3	2	8^*^
MTX	2	2	2	6^*^
RH	2	2	1	5
RH-MTX	2	2	1	5
MTX-SLN	0	1	1	2^*#*^
RH-SLN	1	1	1	3^*#*^
RH-MTX-SLN	0	1	0	1^*#*^

Three fields per each joint were assessed and data presented as mean, *n* = 4. The grades were then summed up into a total score out of 9. Symbols show a comparison to * negative control (NC), and # positive control (AA), using Kruskal–Wallis Test (*p* < 0.05). MTX: methotrexate solution, RH: rhein solution, RH-SLNs: rhein loaded on solid lipid nanoparticles, MTX-SLNs: methotrexate loaded on solid lipid nanoparticles, RH-MTX-SLNs: combination of methotrexate and rhein loaded on solid lipid nanoparticles

## Discussion

The current study was designed to incorporate RH with MTX in targeted solid lipid nanoparticles (RH-MTX-SLNs) aiming at enhancing their bioavailability and utilizing a lower dose of MTX. Different studies have confirmed the advantages of SLNs as a low toxicity, high drug loading, especially for lipophilic drugs, efficient drug targeting, and controlled drug release (Banerjee and Pillai 2019; Campos et al. 2020). Moreover, SLNs have proven efficacy in enhancing lipophilic drugs' absorption and bioavailability (Potta et al. 2010; Yang et al. 2016; Moradpour and Barghi 2019).

In-vitro characterization of the prepared SLNs revealed their occurrence in the nanosize range with high negative ZP indicating their stability. The negative charge might be caused by the slightly ionized fatty acids from Precirol (glyceryl palmitostearate) (Huang et al. 2008).

An increase in the size after drug loading indicates the successful drug entrapment which is further confirmed by % EE results. RH-MTX-SLNs showed the highest particle size, however, still in the nanosize range. This might be due to the incorporation of both RH and MTX in the same SLNs. From the obtained TEM micrographs for MTX-SLNs and RH-MTX-SLNs; a dense layer was revealed surrounding the SLNs. This might indicate that MTX is attached to the outer part of the nanoparticles (Garg et al. 2016). However, RH-SLNs micrographs indicated that the drug is entrapped inside the nanoparticles. This was confirmed by the larger particle size of MTX-SLNs, and the slightly higher zeta potential compared to placebo SLNs and RH-SLNs. Generally, the smaller particle size obtained from TEM micrographs compared to that obtained by Zetasizer might be due to the dehydration of the lipid nanoparticles during sample preparation for TEM (El-Salamouni et al. 2015). The high % EE for both RH and MTX was expected due to their low water solubility which aids their partitioning in the lipid phase. This result was in agreement with other studies using Precirol and Lecinol where they can incorporate large amounts of drugs in their matrix (Feng et al. 2017). Additionally, the use of RH and MTX in small doses and the high lipid: drug ratio help their better entrapment. This was further confirmed by the absence of characteristic drug peaks in the physical mixture and final SLNs formulation FTIR spectra. This indicates the solubility and good dispersion of RH and MTX in the lipids. Additionally, the obtained FTIR spectra revealed the identity of RH, MTX and all the examined excipients.

The obtained release profiles revealed higher RH and MTX release rates from the prepared SLNs compared to suspension. This might be attributed to the solubility of the drugs in the lipid phase and their presence in the amorphous state (Ebada et al. 2021). This significantly enhanced the rate and extent of drug release compared to suspension. The sustained release pattern of the drugs from RH-SLNs, MTX-SLNs and RH-MTX SLNs might be due to the diffusion of RH and MTX from the lipid phase. This sustained release property might enhance drugs absorption from the gastrointestinal tract and allow the drugs to reach their target site. The higher release rate of MTX compared to RH might be due to MTX adsorption onto or below the SLNs surface (Garg et al. 2016). This was clarified in the TEM micrograph as MTX-SLNs and RH-MTX-SLNs showed a dense layer on the outer surface of the SLNs. However, the RH-SLNs TEM micrograph revealed RH loading within the SLNs matrix. MTX release pattern didn't significantly (*P* > 0.05) change upon combining RH and MTX on the same formulation (RH-MTX-SLNs). On the other hand, RH release slightly decreased from RH-MTX-SLNs compared to RH-SLNs. This might be due to the adsorption of MTX on the surface of SLNs.

All the prepared SLNs showed good stability upon storage for 3 months at 4 °C. This might be attributed to the addition of poloxamer 188. This surface active agent increases the repulsion force between the particles reducing their surface free energy and aggregation and hence enhancing their stability (Tanvir and Qiao 2012).

As an autoimmune chronic inflammatory disease, RA is characterized by elevated inflammatory parameters, joint swelling, pain, tenderness and cartilage as well as bone destruction (Smolen et al. 2016). Treatment with RH-MTX-SLNs in this study has reduced all signs of inflammation, manifested as decreased hind paw swelling, mean width of the tibiotarsal joint, improved arthrogram score and a clear decrease in the inflammatory parameters TNF-α and IL-1β. Such effect is attributed to the known anti-inflammatory mechanisms of both RH and MTX in the RA (Cong et al. 2012b; UPADHYAY et al. 2021; Saleem et al. 2022) and confirmed by the histopathology examination in the current study. Interestingly, the combination of RH and MTX without incorporation in the SLNs was not successful in decreasing the TNF-α level; however, the IL-1β was decreased. Knowing that the MTX dose used with RH solution in combination is below the therapeutic dose, the former outcome might be related to the effect of RH on the IL-1β production system. As illustrated by Pelletier et al. (Martel-Pelletier and Pelletier 2010), RH is able to reduce the production of the IL-1 converting enzyme at the cell membrane and consequently reduces the release of the active cytokine IL-1β, besides reducing the number of IL-1 receptors available. Combining both drugs in SLNs allows the drugs to reach the site of action in a higher dose (Bayón-Cordero et al. 2019), this improved drug delivery is then reflected in the effective decrease in TNF-α level, as well as IL-1β.

Impaired apoptosis of synovial cells and inflammatory cells has been a key mechanism in the pathogenesis of RA, leading to synovial cell activation and chronic inflammation together with hyperplasia. Resistance of the synovial fibroblasts to apoptosis has been the hallmark of the progressive destruction of the articular cartilage (Baier et al. 2003). Results of the current study came to confirm the impaired apoptosis in untreated AA rats, showing a high Bcl-2/Bax ratio. In the last years, ERS has shown strong involvement in mediating apoptotic signaling pathways and its activation has been associated with several chronic autoimmune inflammatory diseases (Park et al. 2014). Inflammatory cytokines in the synovium, like IL-1 β and TNF-α, are also known to induce ERS (Zhang et al. 2006). Increased ERS causes disruption in critical ER functions, consequently, some adaptive response proteins get activated to induce repair. One of the repair mechanisms of ER is decreasing a load of proteins, increasing the CHOP that helps in the correction of folded proteins, and increasing the ATF 6. However, in cases of chronic stress, this mechanism fails and a cell death response is induced in form of an apoptosis (Rahmati et al. 2018). This explains the increased expression of both parameters in the AA untreated rats.

It is still not clear whether treatment should increase or decrease ERS to induce apoptosis in RA. In the current study, all treated groups were able to decrease the expression of the known marker of ERS, CHOP, and the sensor of ERS response, ATF 6. This was clearly associated with an increase in Bax level and a decrease in the Bcl-2/Bax ratio promoting apoptosis. The ability of MTX or RH to induce apoptosis was previously proven in other contexts (Ge et al. 2011; Li et al. 2012; Florou et al. 2013; Hassanshahi et al. 2019). Similarly, the results of Rong et al. revealed that MTX treatment was able to decrease signs of ERS (Rong et al. 2018). Treatment with RH has also shown decreased ATF 6 expression levels in an animal model of adjuvant arthritis in a dose-dependent manner. The sharp decrease of the CHOP and ATF 6 expression by the RH-MTX-SLNs indicates a dramatic relief of the adjuvant-induced inflammatory stress and confirms the involvement of ERS in the apoptotic pathway of RA (Cong et al. 2012b). Results of the electron microscope and H&E staining came to confirm the massive improvement of inflammatory infiltrates and synovial hyperplasia of RH-MTX-SLNs showing regeneration of cartilaginous surface.

## Conclusion

In conclusion, RH-MTX-SLNs were successfully prepared with high efficacy and stability. They are involved in the suppression of signaling pathways of anti-apoptotic molecules. Being able to cause a dramatic relief of the adjuvant-induced inflammatory stress, together with regeneration in the cartilaginous surface, nominates it as a therapeutic option for patients with active RA.

### Supplementary Information

Below is the link to the electronic supplementary material.

Supplementary file: 1 (DOCX 30KB)

## Data Availability

All data generated or analyzed during this study are included in this published article and its supplementary information files.
