# Supplementary material for: Rhein methotrexate-decorated solid lipid nanoparticles altering adjuvant arthritis progression through endoplasmic reticulum stress-mediated apoptosis
Source: Inflammopharmacology. 2023 Aug 1;31(6):3127–42. doi: 10.1007/s10787-023-01295-w (PMC10692035; doi:10.1007/s10787-023-01295-w)
Supplement: Supplementary file 1 — Supplementary file: 1 (DOCX 30KB) [file 10787_2023_1295_MOESM1_ESM.doc]

**Table 1:** Primers sequences used for gene expression

| Gene | Primer sequences |
| --- | --- |
| ATF-6 | F: 5'-ATTACTCACCGATCCGAGTT-3' |
|  | R: 5'-CATGAGGAAGAACCGGACTA-3' |
| CHOP | F: 5'-AAGGAAATGCCAGTACCTCA-3' |
|  | R: 5'-CAGGAGCCTGGATTGACAT-3' |
| β-actin | F: 5'-ATGTGGCTGAGGACTTTGATT-3' |
|  | R: 5'-ATCTATGCCGTGGATACTTGG-3' |
